# Association between Gastric Acid Suppression Therapy and Scattered White Spots in the Duodenum: A Retrospective Observational Study

**DOI:** 10.1055/a-2923-5524

**Published:** 2026-08-07

**Authors:** Mamoru Ito, Masakuni Kobayashi, Akira Dobashi, Takanori Tominaga, Ryuta Fujii, Shunsuke Uchiyama, Maki Saihara, Keiko Shimoyama, Naoya Tada, Naoto Tamai, Machi Suka, Kazuki Sumiyama

**Affiliations:** 1 Department of EndoscopyThe Jikei University School of MedicineTokyoJapan; 2 Department of Public Health and Environmental Medicine 12839 The Jikei University School of MedicineTokyoJapan

**Keywords:** endoscopy upper GI tract, reflux disease, ulcers (peptic and other)

## Abstract

**Background and Study Aims** Endoscopic duodenal findings remain undescribed after long-term use of antacids despite their known effects on duodenal physiology. We identified scattered white spots (SWSs) as an endoscopic finding of chronic acid suppression in the duodenum and aimed to confirm this association.

**Patients and Methods** In this single-center, two-arm, retrospective study, we reviewed 200 patients who underwent outpatient esophagogastroduodenoscopy: 100 consecutive patients receiving continuous acid-suppression (proton pump inhibitors [PPI] or potassium-competitive acid blockers [P-CAB] ≥ 8 weeks) and 100 consecutive patients with no history of acid-suppression therapy. Two blinded expert endoscopists independently reviewed all white-light endoscopic images of the descending duodenum. SWSs were graded 0 (absent/minimal), 1 (present) or 2 (dense). SWS positive was defined as a combined score ≥2 of the two independent evaluators.

**Results** A total of 648 images from 200 patients (mean age 62 years, 62.5% male) were analyzed. The acid-suppression group included 58 PPI users and 42 P-CAB users. The SWS-positive rates were significantly higher in the acid-suppression group than the non-suppression group (47% vs. 15%; OR 4.33; 95% confidence interval [CI], 2.20−8.50; *P* < 0.0001). Interobserver agreement was substantial (weighted κ = 0.77; 95% CI, 0.68−0.85). Multivariate logistic regression analysis revealed acid-suppression therapy as an independent factor for SWS (adjusted OR 6.10; 95% CI, 2.89−13.7; *P* < 0.0001).

**Conclusions** Long-term gastric acid suppression is significantly associated with the presence of duodenal SWS. Duodenal SWS associated with PPIs and P-CABs are under-recognized, and further studies are warranted to determine their clinical significance.

## Introduction

Proton pump inhibitors (PPIs) and potassium-competitive acid blockers (P-CABs) are increasingly prescribed for acid-related disorders, including peptic ulcer disease, gastroesophageal reflux disease (GORD), functional gastrointestinal disorders, and prophylaxis of nonsteroidal anti-inflammatory drug (NSAIDs)-associated ulcers. 1 Their superior gastric acid suppression and symptomatic relief compared to histamine-2 receptor antagonists (H2RAs) 2 often prompt their long-term prescription. Although much of the current evidence is observational, concerns regarding the over-prescription have emerged, such as potential risks of malabsorption, infections, and gastric neoplasia. 3 4 5 6 Long-term gastric acid suppression is associated with morphological changes of the gastric mucosal abnormalities on esophagogastroduodenoscopy (OGD), including cobblestone-like appearance, hyperplastic polyps and enlarged fundic gland polyps. 7 8 9 10 11 12 13 14 A series of studies also suggests gastric acid suppression could affect the duodenum, inducing microbiome dysbiosis and mucosal permeability. 15 16 17 18 19 However, the relationship between chronic acid-suppressive therapy and endoscopic duodenal mucosal morphological changes remain unknown. During a preliminary review of duodenal images from patients on long-term acid-suppression therapy, we noticed an increase of “scattered white spots” (SWSs), which are minute, discrete, whitish lesions diffusely distributed throughout the duodenum, predominantly in the second portion. 20 21 To confirm this observation, we conducted a retrospective study with independent review of endoscopic images by blinded experts, to evaluate whether individuals receiving long-term PPI or P-CAB therapy are associated with a higher presence of SWS compared to controls.

## Patients/Material and Methods

### Study Design

This retrospective, single-center observational study was conducted at the Jikei University Hospital (Tokyo, Japan). We retrospectively reviewed the electronic medical records of consecutive patients who underwent outpatient OGD between November 2024 and April 2025. The study protocol was approved by the Institutional Ethics Committee of the Jikei University (Approval No. 37-152) and complied with the Declaration of Helsinki and the “Ethical Guidelines of Medical and Biological Research Involving Human Subjects” of Japan. As the study used only existing clinical data and endoscopic images, informed consent was obtained via an opt-out process, as announced on the hospital’s website.

### Eligibility Criteria

The inclusion criteria were: (i) age ≥18 years; (ii) routine outpatient OGD performed using the GIF-H290 endoscope (Olympus Medical, Tokyo, Japan) at the Jikei University Hospital; and (iii) at least one documented white-light image of the second portion of the duodenum. Patients were excluded if they met any of the following criteria: (i) inadequate imaging (e.g., blur, food residue, close-up views, or bubbles) that inhibited sufficient inspection of the duodenal mucosa; (ii) hereditary polyposis syndromes; (iii) inflammatory bowel disease, coeliac disease, Zollinger-Ellison syndrome, or autoimmune gastritis; (iv) ongoing chemotherapy or radiotherapy; (v) prior duodenal surgery; (vi) active *Helicobacter pylori* ( *H. pylori* ) infection; (vii) active duodenal ulcerative or neoplastic lesions; (viii) decompensated liver or heart failure, chronic kidney disease (creatinine ≥ 2.0 mg/dL) or anemia (hemoglobin [Hb] ≤ 9.0 g/dL); (ix) use of NSAIDs or systemic corticosteroids; and (x) request to refuse study participation. The exclusion criteria were designed to rule out medications or systemic conditions, which could induce duodenal inflammation, ulcerations, erosions, or edema. Patients with active *H. pylori* infection were also excluded to prevent confounding, as previous reports have demonstrated its association with SWS. 21

### Allocation

Patients were categorized into two groups based on the medication history documented in their electronic medical records. The acid-suppression group was defined as individuals receiving PPIs or P-CABs continuously for ≥8 weeks at the time of OGD. Patients who had received only short-term therapy for peptic ulcer disease were excluded. The non-suppression group consisted of individuals with no recorded use of PPIs, P-CABs, or H2RAs at the time of OGD.

### Review of Electronic Medical Record

A list of routine OGDs performed at the Jikei University Hospital was extracted using the CLISTA! data warehouse system (Medical Engineering Institute, Inc., Tsu, Japan). For the acid-suppression group, potential candidates were identified by searching for the keywords “vonoprazan,” “lansoprazole,” “omeprazole,” “esomeprazole,” and their respective commercial brand names during the study period. For the non-suppression group, the list was extracted by excluding the keywords “vonoprazan,” “lansoprazole,” “omeprazole,” “esomeprazole,” and “famotidine” along with their respective brand names. Each electronic medical record was manually reviewed to confirm eligibility and to extract the following data: age, sex, height, weight, medical history, smoking and alcohol consumption history, current medications, *H. pylori* status, endoscopic diagnoses, and laboratory results. Laboratory data included aspartate transaminase, alanine aminotransferase, albumin, estimated glomerular filtration rate, C-reactive protein, white blood cell count, Hb, HbA1c, serum glucose, triglyceride, high-density lipoprotein cholesterol, low-density lipoprotein cholesterol, and eosinophil counts, all obtained within 6 months of the OGD. To minimize selection bias, consecutive OGD cases were reviewed until the target sample size of eligible patients was enrolled.

### Endoscopic Image Assessment of SWS

**Fig. 1:**
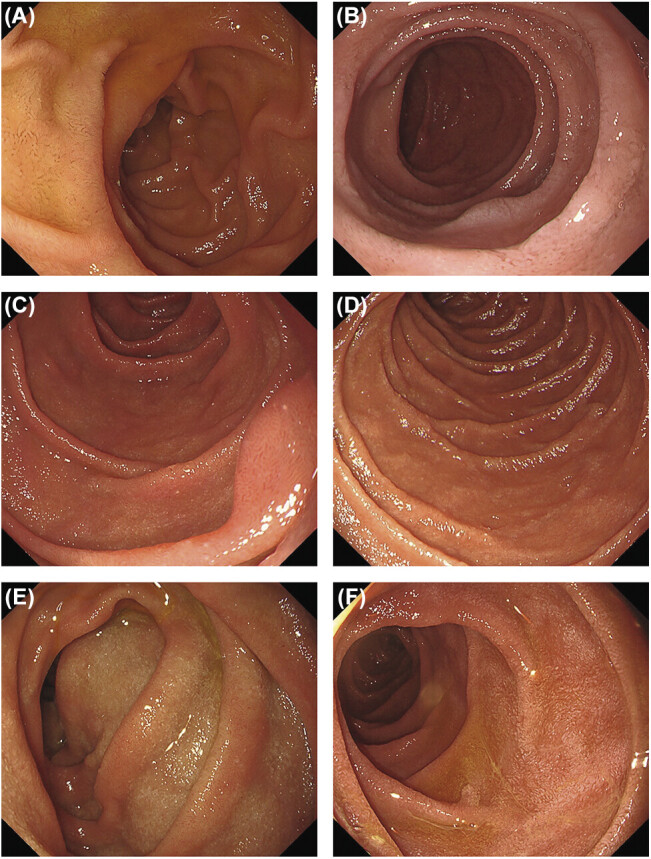
Sample images of duodenal scattered white spots (SWSs) grading system. ( **A, B** ) Sample images of Grade 0. Grade 0 is defined as no scattered white spots or detection requires careful examination. ( **C, D** ) Sample images of Grade 1. Grade 1 is defined as mild but immediately apparent white spots. ( **E, F** ) Sample images of Grade 2. Grade 2 is defined as dense appearance of white spots in duodenal mucosa.

For each patient, all still images of the descending duodenum obtained under white-light imaging were exported from the SOLEMIO QUEV (Olympus Medical Co., Tokyo, Japan) in anonymized PNG format and stored in a dedicated study database. A correspondence table linking study identifiers with patient information was kept separately. All images were independently reviewed by two board-certified endoscopists, each with over 15 years of experience (AD, MK), who were blinded to all clinical data and group allocation. SWSs were graded for each patient according to the following prespecified criteria: Grade 0, no or minimal SWS ( Fig. 1A,B ); Grade 1, mild but immediately recognizable SWS ( Fig. 1C,D ); and Grade 2, densely clustered SWS ( Fig. 1E,F ). Reference sample images ( Fig. 1A–F ) were provided to the reviewers to support evaluation. For patients with multiple white-light images of descending duodenum, the reviewing endoscopists performed an assessment of all available images to assign a single grade that best represented the maximum density of SWS observed in each patient. The combined score from the two reviewers (range 0–4) was calculated for each patient, and SWS positive was defined as a combined score of ≥2, while a combined score <2 was considered SWS negative.

### Outcomes

The primary endpoint was the SWS-positive rate, defined as the proportion of SWS-positive patients in each group. Secondary endpoints included: (i) interobserver agreement for SWS grading, (ii) the proportion of cases with high-grade SWS (combined score of 3 or 4) among SWS-positive patients; (iii) the SWS-positive rates compared between PPI and P-CAB users; and (iv) SWS-positive rates stratified by age, sex, body-mass index, smoking status, alcohol consumption, past medical history, *H. pylori* status, dose or duration of acid-suppressive therapies, endoscopic findings, and laboratory findings.

### Statistical Methods

Sample size was determined based on pilot data from PPI/P-CAB users who underwent OGD at May 2025, where 8 out of 36 (22%) patients exhibited SWS of ≥Grade 1 as assessed by a board-certified endoscopist. Given that the SWS-positive rate in the general population has been reported as 3.2%, 21 we conservatively assumed a SWS-positive rate of 20% in the acid-suppression group and 5% in the non-suppression group. A sample size of 100 patients per group (total *n* = 200) was required to achieve 90% statistical power with a two-sided α of 0.05.

Standard descriptive statistics were used to calculate medians, ranges and frequencies. Non-normally distributed variables were reported as median (interquartile range) and normally distributed variables as mean (±standard deviations). Categorical variables were compared using the chi-squared test or Fisher’s exact test when expected counts were <5. Continuous variables were analyzed using Student’s *t* test or the Mann–Whitney *U* test, as appropriate. A two-sided *P* -value < 0.05 was considered statistically significant. To identify factors independently associated with SWS positivity, univariable and multivariable logistic regression analyses were performed. Variables for multivariable model were selected based on baseline differences between the acid-suppression group and the non-suppression group, as well as between SWS-positive patients and SWS-negative patients. Adjusted odds ratios (ORs) with 95% confidence intervals (CIs) were calculated. Interobserver agreement for SWS grading was evaluated using weighted κ statistics. Cases with missing data were excluded from the analysis, and no imputation was performed. All statistical analyses were conducted using R (version 4.3.2, The R Foundation, Vienna, Austria).

## Results

**Fig. 2:**
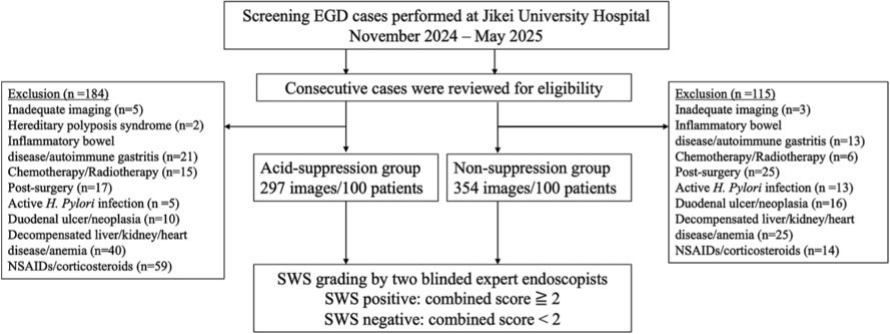
Flowchart of patients through enrollment, allocation and analysis. Abbreviations: *H. pylori* , *Helicobacter pylori* ; NSAIDs, nonsteroidal anti-inflammatory drugs; OGD, esophagogastroduodenoscopy; SWS: scattered white spots.

Consecutive cases were reviewed in reverse chronological order until 100 patients meeting eligibility criteria were included in each group. A total of 499 consecutive OGD cases were screened ( Fig. 2 ). In the acid-suppression group, 184 cases were excluded for the following reasons: inadequate imaging ( *n* = 5); hereditary polyposis syndromes ( *n* = 2); inflammatory bowel disease or autoimmune gastritis ( *n* = 21); ongoing chemotherapy or radiotherapy ( *n* = 15); prior duodenal surgery ( *n* = 17); active *H. pylori* infection ( *n* = 5); active duodenal ulcerative or neoplastic lesions ( *n* = 10); decompensated liver disease, chronic kidney disease or anemia ( *n* = 40); and use of NSAIDs or systemic corticosteroids ( *n* = 69). In the non-suppression group, 115 cases were excluded due to: inadequate imaging ( *n* = 3); inflammatory bowel disease or autoimmune gastritis ( *n* = 13); ongoing chemotherapy or radiotherapy ( *n* = 6); prior duodenal surgery ( *n* = 25); active *H. pylori* infection ( *n* = 13); active duodenal ulcerative or neoplastic lesions ( *n* = 16); decompensated liver disease, chronic kidney disease, or anemia ( *n* = 25); and the use of NSAIDs or systemic corticosteroids ( *n* = 14). Consequently, 294 images from 100 patients in the acid-suppression group and 354 images from 100 patients in the non-suppression group were included in the final analysis.

### Patient Characteristics

Baseline characteristics of the study population are summarized in Table 1 . Patients in the acid-suppression group were significantly older (median 66.0 vs. 59.0 years, *P* = 0.038) and included a higher proportion of males (73% vs. 52%, *P* = 0.002). BMI was also slightly higher in the acid-suppression group (23.7 vs. 22.3 kg/m ^2^ , *P* = 0.008). Smoking and alcohol consumption histories differed between the groups, largely due to a high proportion of unknown data. Several comorbidities were more prevalent among acid-suppression users, including type 2 diabetes mellitus (T2DM; 17% vs. 4%, *P* = 0.0027), GORD (40% vs. 9%, *P* < 0.0001), dyslipidemia (20% vs. 10%, *P* = 0.048) and metabolic dysfunction-associated steatotic liver disease (MASLD; 8% vs. 0%, *P* = 0.048). Endoscopic findings of hiatal hernia (31% vs. 13%, *P* = 0.002) and hyperplastic polyps (16% vs. 0%, *P* < 0.0001) were also significantly more frequent in the acid-suppression group. Additionally, the use of statins (22% vs. 5%, *P* = 0.0004) and antidiabetic agents (17% vs. 3%, *P* < 0.0001) was significantly more common in acid-suppression users.

**Table 1:** Baseline characteristics of enrolled patients.

	Acid-suppression group ( *n* = 100)	Non-suppression group ( *n* = 100)	*P* -value
Age; years, median (IQR)	66.0 (56.3−74.0)	59.0 (52.0−72.0)	0.038*
Sex; male/female	73/27	52/48	0.0022**
BMI; kg/m ^2^ , median (IQR)	23.7 (21.0−26.4)	22.3 (19.9−24.6)	0.0081*
Smoking history; *n* (%) Never smoker Past/current smoker Unknown	14 (14) 33 (33) 53 (53)	33 (33) 29 (29) 38 (38)	0.0055**
Drinking history; *n* (%) Never drinker/occasional Daily (≥3/week) Unknown	8 (8) 27 (27) 65 (65)	26 (26) 35 (35) 39 (39)	0.0002**
PPI/P-CAB prescription P-CAB Vonoprazan 20 mg Vonoprazan 10 mg Total PPI Esomeprazole 20 mg Esomeprazole 10 mg Lansoprazole 30 mg Lansoprazole 15 mg Rabeprazole 20 mg Rabeprazole 10 mg Total Length of prescription <6 months 6–12 months 1–3 years 3–5 years 5–10 years 11–20 years	17 (17) 25 (25) 42 (42) 18 (18) 10 (10) 4 (4) 20 (20) 4 (4) 2 (2) 58 (58) 5 (5) 8 (8) 21 (21) 22 (22) 12 (12) 32 (32)	N/A	N/A
OGD indication; *n* (%) Follow-up/screening GORD Symptomatic	60 (60) 28 (28) 12 (12)	86 (86) 6 (6) 8 (8)	<0.0001**
Past medical history; *n* (%) Type 2 Diabetes Mellitus Hypertension Dyslipidemia MASLD GORD Post-cholecystectomy	17 (17) 28 (28) 20 (20) 8 (8) 40 (40) 7 (7)	4 (4) 17 (17) 10 (10) 0 (0) 9 (9) 3 (3)	0.0027** 0.062** 0.048** 0.0068*** <0.0001** 0.19***
*H. pylori* status; *n* (%) Uninfected Eradicated	67 (67) 33 (33)	58 (58) 42 (42)	0.24**
Endoscopic findings; *n* (%) GORD Hiatal hernia Barrett’s esophagus Fundic gland polyp Hyperplastic polyp Atrophic gastritis	26 (26) 31 (31) 27 (27) 47 (47) 16 (16) 37 (37)	24 (24) 13 (13) 18 (18) 37 (37) 0 (0) 46 (46)	0.74** 0.002** 0.13** 0.15** <0.0001** 0.20**
Medications; % ARB/ACEI Calcium channel blockers Statins Probiotics Antidiabetics	18 (18) 19 (19) 22 (22) 8 (8) 17 (17)	11 (11) 12 (12) 5 (5) 2 (2) 3 (3)	0.16** 0.17** 0.0004** 0.052** 0.0015***

Abbreviations: ARB/ACEI, angiotensin II receptor blocker/angiotensin-converting enzyme inhibitor; BMI, body mass index; DM, diabetes mellitus; GORD, gastroesophageal reflux disease; MASLD, metabolic dysfunction-associated steatotic liver disease; N/A, not available; P-CAB, potassium-competitive acid blockers; PPI, proton pump inhibitor; SWS, scattered white spots. *Mann–Whitney, **chi-squared, ***Fisher’s exact.

Among the 100 patients receiving acid-suppression therapy, 58 were taking PPIs and 42 were taking P-CABs at the time of OGD. Vonoprazan was the most frequently prescribed agent, administered at a dose of 20 mg in 17 patients and 10 mg in 25 of patients. Esomeprazole was prescribed at 20 mg ( *n* = 18) and 10 mg ( *n* = 10); lansoprazole at 30 mg ( *n* = 4) and 15 mg ( *n* = 20), and rabeprazole at 20 mg ( *n* = 4) and at 10 mg ( *n* = 2). The duration of therapy was as follows: <6 months ( *n* = 5), 6–12 months ( *n* = 8), 1–3 years ( *n* = 21), 3–5 years ( *n* = 22), 5–10 years ( *n* = 12), and 11–20 years ( *n* = 32).

### SWS-Positive Rates and Grading

**Fig. 3:**
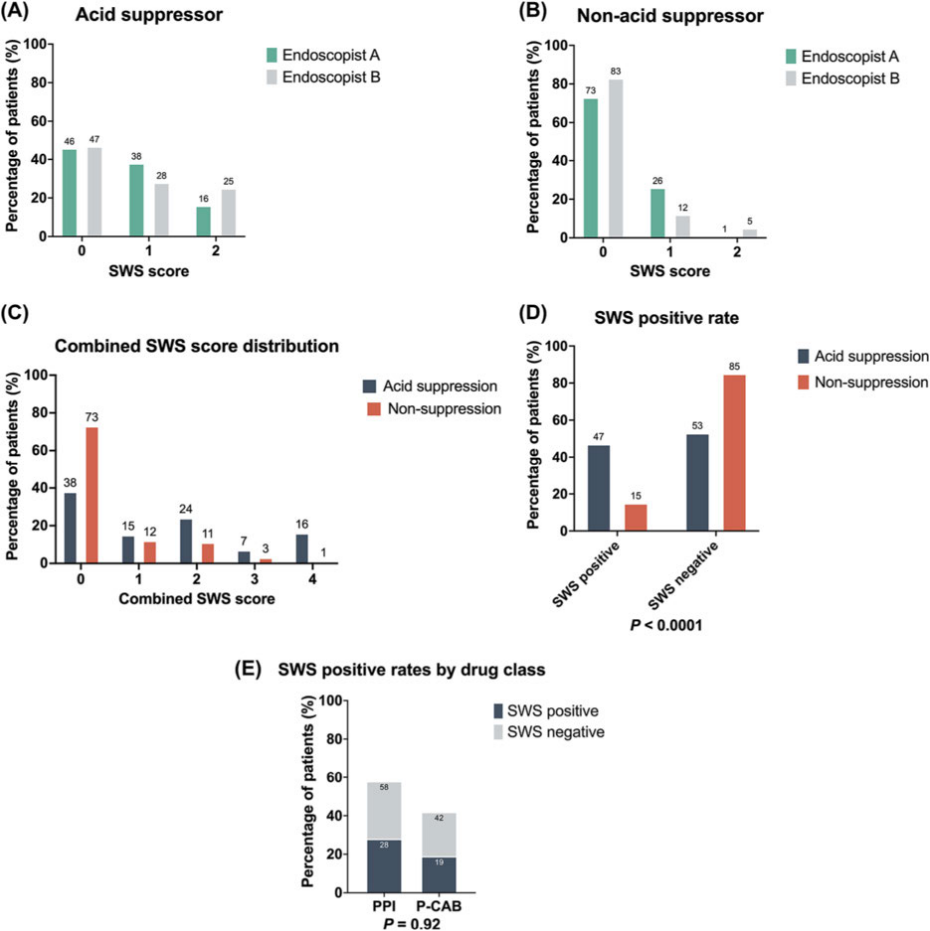
Quantitative analysis and interobserver agreement of duodenal scattered white spots (SWS). ( **A, B** ) Interobserver agreement for SWS grading in the acid-suppression group ( **A** ) and non-suppression ( **B** ) groups. Overall weighted κ was 0.77 (95% CI 0.68−0.85). ( **C** ) Distribution of combined SWS scores (0–4). ( **D** ) SWS-positive rate (combined score ≥2) was significantly higher in the acid-suppression group ( *P* < 0.0001). ( **E** ) SWS-positive rate by drug class (PPI vs. P-CAB). Numbers above or within bars represent patient counts. Abbreviations: CI, confidence interval; P-CAB, potassium-competitive acid blocker; PPI, proton pump inhibitor; SWS, scattered white spots.

Among the 200 included patients, 62 (31%) were classified as SWS-positive, defined by combined score of ≥2. In the acid-suppression group, the two endoscopists assigned identical grades in most cases: for Grade 0, *n* = 46 versus 47; for Grade 1, *n* = 38 versus 28; and for Grade 2, *n* = 16 versus 25 ( Fig. 3A ). Similarly, in the non-suppression group, a high degree of consistency was observed, with both reviewers grading most patients as Grade 0 ( *n* = 73 vs. 83) ( Fig. 3B ). Overall, the interobserver agreement for SWS grading was substantial, with a weighted κ value of 0.77 (95% CI, 0.68−0.85). The SWS-positive rates were significantly higher in the acid-suppression group than in the non-suppression group (47% vs. 15%; OR 4.33, 95% CI 2.20−8.50; *P* < 0.0001) ( Fig. 3C ). The distribution of SWS scores also differed between the groups; the acid-suppression group demonstrated a lower proportion of score 0 (38% vs. 73%) and a higher proportion of score 4 (16% vs. 1%) compared to the non-suppression group ( Fig. 3D ).

### Comparison of SWS-positive and SWS-negative patients

characteristics stratified by SWS status are summarized in Table 2 . Age, sex, BMI, smoking, or alcohol consumption histories did not differ significantly between patients with and without SWS. Regarding comorbidities, only GORD was more frequent in SWS-positive patients (36% vs. 20%, *P* = 0.02), while no significant associations were found for T2DM, hypertension, dyslipidemia, and MASLD were not significantly associated. OGD findings of hyperplastic polyps (15% vs. 5%, *P* = 0.044) and hiatal hernia (31% vs. 18%, *P* = 0.048) were more common in SWS-positive patients. Meanwhile, atrophic gastritis was more frequent in SWS-negative patients (31% vs. 46%, *P* = 0.037). The use of major medication classes did not differ significantly, although a trend toward higher angiotensin II receptor blocker/angiotensin-converting enzyme inhibitor (ARB/ACEI) use was observed in SWS-positive patients (21% vs. 12%, *P* = 0.082). SWS-positive rates did not differ between PPI and P-CAB users (28/58 [48%] vs. 19/42 [45%], *P* = 0.92; Fig. 3E ). *H. pylori* infection status (31% vs. 41%, *P* = 0.23) and major laboratory values showed no significant differences between the two groups.

**Table 2:** Comparison of clinical characteristics and endoscopic findings between SWS-positive and SWS-negative patients.

	SWS-positive ( *n* = 62)	SWS-negative ( *n* = 138)	*P* -value
Age; years, median (IQR)	65.0 (53.0−74.3)	61.0 (54.8−73.0)	0.35*
Sex; male/female	41/21	84/54	0.48**
BMI; kg/m ^2^ , median (IQR)	22.5 (20.0−24.7)	23.1 (20.6−25.8)	0.36*
Smoking history; *n* (%) Never smoker Past/current smoker Unknown	16 (26) 20 (32) 26 (42)	31 (22) 42 (30) 65 (47)	0.78**
Drinking history; *n* (%) Never drinker/occasional Daily (≥3/week) Unknown	12 (19) 19 (31) 31 (50)	22 (16) 43 (31) 73 (53)	0.83**
Past medical history; *n* (%) Type 2 DM Hypertension Dyslipidemia MASLD GORD Cholecystectomy	8 (13) 17 (27) 9 (15) 5 (8) 22 (36) 3 (5)	13 (9) 28 (20) 20 (14) 3 (2) 27 (20) 14 (10)	0.23** 0.26** >0.99** 0.11*** 0.02** 0.21**
*H. pylori* status; *n* (%) Uninfected Eradicated	43 (69) 19 (31)	82 (59) 56 (41)	0.23**
Endoscopic findings; *n* (%) GORD Hiatal hernia Barrett’s esophagus Fundic gland polyp Hyperplastic polyp Atrophic gastritis	14 (23) 19 (31) 16 (26) 20 (32) 9 (15) 19 (31)	36 (26) 25 (18) 29 (21) 64 (46) 7 (5) 64 (46)	0.60** 0.048** 0.45** 0.061** 0.044*** 0.037**
PPI/P-CAB; *n* (%) P-CAB PPI	19 (31) 28 (45)	23 (17) 30 (22)	0.92**
Length of prescription; *n* (%) <1 year 1–5 years 5–10 years 11–20 years	6 (10) 21 (34) 7 (11) 13 (21)	7 (5) 22 (16) 5 (4) 19 (14)	0.75**
Medications; *n* (%) ARB/ACEI Calcium channel blockers Statins Probiotics Antidiabetics	13 (21) 10 (16) 9 (15) 5 (8) 8 (13)	16 (12) 21 (15) 18 (13) 5 (4) 12 (9)	0.082** 0.87** 0.78** 0.29*** 0.36**
Laboratory findings AST; U/L, median (IQR) ALT; U/L, median (IQR) Alb; g/dL, median (IQR) eGFR; mL/min/1.73 m ^2^ (IQR) CRP; mg/dL (IQR) WBC; 10 ^3^ /μL Hb; g/dL (IQR) HbA1c; % (IQR) Glu; mg/dL (IQR) TG; mg/dL (IQR) HDL-C; mg/dL (IQR) LDL-C; mg/dL (IQR) Eos; % (IQR)	22.5 (19.3−28.0) 18.5 (14.3−24.0) 4.2 (4.0−4.4) 70.5 (58.5−82.0) 0.05 (0.04−0.13) 5,400 (4,600−6,400) 14.1 (12.9−14.9) 5.9 (5.6−6.3) 102 (95−125) 113 (70−162) 57 (51−82) 117 (102−132) 1.7 (0.6−4.1)	21.0 (18.0−25.6) 17.0 (14.0−24.0) 4.2 (4.0−4.5) 73.0 (61.0−84.0) 0.06 (0.04−0.12) 5,400 (4,500−6,400) 13.7 (12.7−15.0) 5.8 (5.7−6.3) 106 (94−122) 120 (67−181) 62 (55−80) 117 (105−148) 1.7 (0.4−3.4)	0.17* 0.82* 0.69* 0.64* 0.90* 0.79* 0.34* 0.90* 0.80* 0.97* 0.42* 0.29* 0.61*

Abbreviations: Alb, albumin; ALT, alanine aminotransferase; ARB/ACEI, angiotensin II receptor blocker/angiotensin-converting enzyme inhibitor; AST, aspartate transaminase; BMI, body mass index; CRP, C-reactive protein; DM, diabetes mellitus; eGFR, estimated glomerular filtration rate; Eos, eosinophil counts; GORD, gastroesophageal reflux disease; Glu, serum glucose; Hb, hemoglobin; HbA1c, hemoglobin A1c; HDL-C, high-density lipoprotein cholesterol; IQR, interquartile range; LDL-C, low-density lipoprotein; MASLD, metabolic dysfunction-associated steatotic liver disease; OR, odds ratio; P-CAB, potassium-competitive acid blockers; PPI, proton pump inhibitor; SWS, scattered white spots; TG, triglyceride; WBC, white blood cell count. *Mann–Whitney test, **chi-squared test, ***Fisher’s exact test.

### Multivariable Logistic Regression Analysis

The multivariable model included acid suppression, age, sex, BMI, history of T2DM, atrophic gastritis on OGD, and ARB/ACEI use as covariates. Age, sex, BMI, and history of T2DM were included because baseline differences were observed between the acid-suppression group and the non-suppression group. Dyslipidemia, MASLD, GORD, hiatal hernia, the use of statin, and antidiabetics were excluded due to collinearity with BMI and acid suppression. The inclusion of T2DM was particularly justified by the emerging recognition of duodenal mucosa as a therapeutic target of metabolic regulation. 22 Smoking status and alcohol consumption were excluded since a high proportion of data were missing (>35%). Atrophic gastritis and ARB/ACEI use were included due to marginally statistical difference ( *P* < 0.1) observed between SWS-positive and SWS-negative patients. The results of the logistic regression analysis are presented in Table 3 . Acid suppression remained the sole independent predictor of SWS, with the adjusted OR of 6.10 (95% CI 2.89−13.7; *P* < 0.0001). On the other hand, BMI showed a non-significant trend toward an inverse association, with the adjusted OR of 0.92 (95% CI 0.84−1.00; *P* = 0.064) per 1 kg/m ^2^ increase.

**Table 3:** Multivariable logistic regression analysis for predictors of SWS.

Variable	Adjusted OR	95% CI	*P* -value
Acid suppression	6.10	2.89−13.7	<0.0001
Age (per 1-year increase)	0.99	0.97−1.02	0.53
Male sex	1.03	0.48−2.22	0.94
BMI (per 1 kg/m ^2^ increase)	0.92	0.84−1.00	0.064
Type 2 DM	1.84	0.62−5.55	0.27
Atrophic gastritis	1.06	0.52−2.17	0.88
ARB/ACEI	1.83	0.71−4.70	0.21

Abbreviations: ARB/ACEI, angiotensin II receptor blocker/angiotensin-converting enzyme inhibitor; BMI, body mass index; CI, confidence interval; DM, diabetes mellitus; OR, odds ratio; SWS, scattered white spots.

## Discussion

This retrospective study demonstrated an association between SWS and long-term PPI or P-CAB therapy. Compared to the non-suppression group, the acid-suppression group had a higher SWS-positive rate with greater grading scores (combined scores of 3 and 4). The reliability of these endoscopic evaluations was validated by a high interobserver agreement (weighted κ = 0.77) between two blinded expert endoscopists. Furthermore, multivariate analysis confirmed a strong, independent association between PPIs/P-CABs and duodenal SWS after adjustment for demographic, metabolic and endoscopic variables.

SWS, sometimes referred to as “white villi” or duodenal lymphangiectasia, are observed as fine, diffusely distributed white speckled points aligned with the villous tips of the descending duodenum. 20 21 23 Historically, these findings have been regarded as benign, physiological response to high-fat dietary intake. 24 A study in 1978 demonstrated that an oral olive oil load induced transient SWS in healthy volunteers. 25 Incidentally discovered SWS in patients without clinical symptoms are typically managed with observation alone, requiring no further investigation. It can sometimes manifest as a secondary finding associated with etiologies such as primary intestinal lymphangiectasia, active *H. pylori* infection, chronic non-specific duodenitis, and giardiasis, in which case the underlying cause is treated. 20 21

In the present study, the SWS-positive rate was 47% in the acid-suppression group and 15% in the non-suppression group, both of which are notably higher than the previously reported 3.2%. 21 This difference might be attributed to several methodological factors. First, misinterpretation of light-reflection artifacts as SWS during the retrospective review of endoscopic images could have elevated the SWS-positive rates in both groups. Second, under-recorded PPI/P-CAB use from other institutions that were not documented in our electronic medical records could have increased the SWS-positive rate in the non-suppression group. Finally, potential residual confounding factors such as high-fat dietary intake cannot be ruled out, since the acid-suppression group had a higher prevalence of metabolic comorbidities including higher BMI, T2DM, dyslipidemia, and MASLD.

Meanwhile, a trend of an inverse relationship between BMI and SWS was observed in the multivariable analysis (adjusted OR 0.92 per 1 kg/m ^2^ increase; 95% CI 0.84−1.00; *P* = 0.064). This unexpected finding contrasts with the assumption that it would be associated with higher BMI because of its link to high-fat dietary habit. The inverse trend suggests that the development of SWS does not simply occur from metabolic pathways, and factors unique to individuals with a lower BMI might be correlated with the manifestation of SWS.

A limitation of this study is the lack of pathological analyses, as duodenal biopsies are not routinely performed in standard clinical practice. Preceding histopathological studies of SWS have reported them as dilated lymphatic vessels in the stroma with chylomicrons and precipitated lymph proteins. 26 Because chronic gastric acid suppression has been associated with various physiological changes including reduced duodenal mucosal permeability, altered lipid-related epithelial transport and dysbiosis, 15 16 17 18 19 we surmise that these factors contribute to the morphological changes and the whitish appearance of the duodenal mucosa. Whether PPIs/P-CABs directly induce these mucosal alterations or act synergistically with other factors remains to be explored.

Recognizing the association between SWS and gastric acid suppression agents could serve as an opportunity to reassess the ongoing therapy. In principle, long-term acid-suppression therapy should be maintained at the minimum effective dose for the shortest possible duration 27 because sustained, excessive acid suppression can induce adverse events. For example, chronic hypergastrinemia from acid suppression can facilitate the growth of hyperplastic polyps, potentially increasing the risk of complications such as gastrointestinal bleeding. 28 29 30 31 32 Our findings of increased gastric hyperplastic polyps in the SWS-positive patients might reflect an early stage of this mucosal alteration. However, the endoscopic findings of SWS alone should not prompt physicians to refrain from prescribing necessary gastric acid suppression. Longitudinal documentation of SWS is needed to clarify their clinical implications. Therefore, rather than relying on a single finding, a comprehensive assessment of the patient’s background and clinical status is recommended.

Several limitations need to be considered. First, the retrospective, single-center design inherently introduces a risk of selection bias. Second, direct measurements of gastric acid output or intraduodenal pH were not performed, precluding any definitive causal inference. Third, a substantial proportions data were missing regarding lifestyle factors such as smoking and alcohol consumption. Finally, the assessment of SWS by the reviewing endoscopists relied on still images of the descending duodenum rather than video assessment. Subtle mucosal changes may have been overlooked, and variations in photographic technique such as distance and light exposure may have influenced the visualization of SWS. In addition, the endoscopic appearance and density of SWS are likely to vary depending on the timing of endoscopy relative to meals and duration of PPI/P-CAB therapy, even within the same patient. 33 34

In conclusion, our study demonstrates a significant association between long-term gastric acid suppression therapy and the presence of SWS in the duodenum. These duodenal alterations remain under-recognized and their clinical implications are yet to be fully understood. Routine documentation of SWS during clinical practice may provide valuable insights. Prospective studies incorporating histopathological and microbiome assessments are required to clarify the underlying mechanisms.
